# Genetic Diversity and Population Structure of Rice Pathogen *Ustilaginoidea* *virens* in China

**DOI:** 10.1371/journal.pone.0076879

**Published:** 2013-09-30

**Authors:** Xianyun Sun, Shu Kang, Yongjie Zhang, Xinqiu Tan, Yufei Yu, Haiyong He, Xinyu Zhang, Yongfeng Liu, Shu Wang, Wenxian Sun, Lei Cai, Shaojie Li

**Affiliations:** 1 State Key Laboratory of Mycology, Institute of Microbiology, Chinese Academy of Sciences, Beijing, P. R. China; 2 School of Life Sciences, Shanxi University, Taiyuan, P. R. China; 3 Institute of Plant Protection, Hunan Academy of Agricultural Science, Changsha, P. R. China; 4 Guizhou Institute of Plant Protection, Guiyang, P. R. China; 5 Institute of Plant Protection, Jiangsu Academy of Agricultural Science, Nanjing, P. R. China; 6 Institute of Plant Protection, Liaoning Academy of Agricultural Science, Shenyang, P. R. China; 7 Department of Plant Pathology, China Agricultural University, Beijing, P. R. China; California Department of Public Health, United States of America

## Abstract

Rice false smut caused by the fungal pathogen *Ustilaginoidea* *virens* is becoming a destructive disease throughout major rice-growing countries. Information about its genetic diversity and population structure is essential for rice breeding and efficient control of the disease. This study compared the genome sequences of two *U* *. virens* isolates. Three SNP-rich genomic regions were identified as molecular markers that could be used to analyze the genetic diversity and population structure of *U* *. virens* in China. A total of 56 multilocus sequence types (haplotypes) were identified out of 162 representative isolates from 15 provinces covering five major rice-growing areas in China. However, the phylogeny, based on sequences at individual SNP-rich regions, strongly conflicted with each other and there were significant genetic differences between different geographical populations. Gene flow between the different geographical populations and genetic differentiation within each geographical population were also detected. In addition, genetic recombination and genetic isolation resulting from geographic separation was also found.

## Introduction

False smut (green smut) is one of the most destructive diseases of rice. It is caused by the ascomycete fungus *Ustilaginoidea* *virens* (Cooke) Takah (Anamorph). *U* *. virens* was recently placed in Clavicipitaceae as the new name *Villosiclava* *virens* (Teleomorph) [1,2], which can reproduce both sexually and asexually with multiple propagules [3,4]. It was found that *U* *. virens* could invade rice coleoptiles and roots at the young seedling stage and the stamen filaments at the earlier booting stage [5-7]. However, it is not clear if *U* *. virens* mycelia, that colonized in young seedlings, can extend to the spikelets and form the ball-like symptom. At rice booting stage, *U* *. virens* invades rice through small gap at the apex of spikelet before heading [6] but does not penetrate host cell walls [7]. The characteristic symptom of rice false smut is the formation of ball-like colonies in spikelets. Sclerotia and chlamydospores are commonly found on the surface of and inside smut balls, respectively. In addition to yield loss, *U* *. virens* also contaminates rice seed and straw by producing antimitotic cyclic peptides that are poisonous to both humans and animals [8,9]. False smut of rice was previously regarded as a minor disease that occurred sporadically in certain regions, but in recent years, the disease has become significant throughout major rice-growing countries [10–12]. The use of nitrogen fertilizers and large-scale planting of hybrid cultivars have been regarded as responsible for the increased disease severity of false smut in China [13].

Previous studies on rice false smut mainly focused on its occurrence, pathogen detection, toxin analysis, pathogen life cycle and disease control [11,14–19]. However, little is known about the genetic diversity of *U* *. virens* populations. Information on the genetic diversity of *U* *. virens* populations will help create efficient strategies to reduce the yield loss caused by this pathogen. Zhou et al. [20] analyzed the genetic diversity of *U* *. virens* populations from two provinces in North China using amplified fragment length polymorphism (AFLP) markers and discovered that the genetic variation in *U* *. virens* populations was small in these areas. However, the samples used for the analysis were collected from only two provinces. The genetic diversity and population structure of *U* *. virens* in the majority of the rice-growing areas across China are unknown.

Methods based on molecular markers have been developed in order to analyze genetic diversity in fungal species. These markers include: allozyme, AFLP and microsatellites. DNA sequence-based markers have been widely used over recent years due to the reduced cost of DNA sequencing. Since an individual gene contains limited information, multi-locus sequence typing (MLST) has become a mainstream method for both molecular systematics and population genetics analyses [21,22]. MLST has been demonstrated to be highly discriminatory when analyzing the genetic diversities of many fungal species that are pathogenic to humans, such as: *Histoplasma capsulatum* [23], *Candida albicans* [24], *Aspergillus fumigatus* [25] and Cryptococcus neoformans [26]. Most of the DNA sequences used for MLST in these studies were limited to housekeeping genes. For plant pathogenic fungi, however, reports on genetic diversity, based on MLST, only found in *Magnaporthe grisea* [27].

Chlamydospores of *U* *. virens* can persist on seeds or in soil for several years [28]. The accumulation of chlamydospores in field or movement of chlamydospores to other areas can increase disease severity. Analysis of genetic diversity in *U* *. virens* isolates from China on a large geographical scale will improve the design of efficient management strategies for rice false smut. In this study, three SNP-rich genomic regions were identified as novel molecular markers that could be used to study the population structure of *U* *. virens* . The genetic diversity and population structure of *U* *. virens* in China were examined by comparing sequences located in SNP-rich genomic regions of 162 *U* *. virens* isolates collected from five major rice-growing areas in China.

## Materials and Methods

Ethics statement: No specific permissions were required for sample collection on the locations mentioned in this manuscript and the fungus used in this study is not an endangered or protected species.

### Collection and isolation of *U* *. virens* isolates

**Figure 1:**
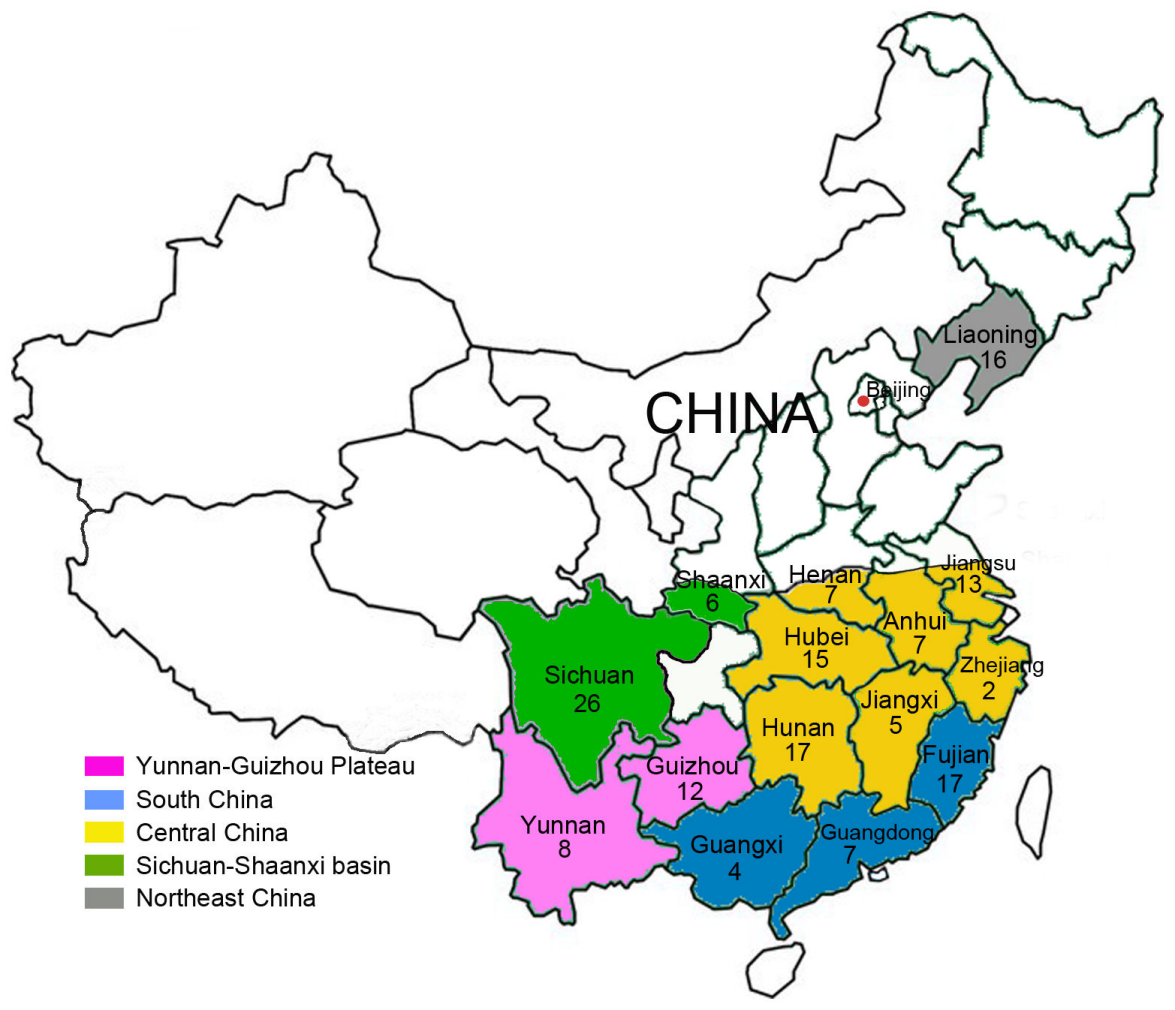
Geographical distributions of *U. virens* samples in China. A total of 162 *U*. *virens* isolates from 15 provinces, covering the five major rice-growing areas of China, were used in this study. Each rice-growing area is marked with a unique color on the map.

All isolates were obtained from infected rice spikelets by single chlamydospore isolation. The *U* *. virens* isolates were recovered from five rice cropping regions (Figure 1; Table S1), based on rice cropping regionalization in China [29], including 32 isolates from Sichuan-Shaanxi basin (Sichuan and South Shaanxi) where single-cropping hybrid rice is cultivated, 28 isolates from South China (Guangdong, Guangxi and Fujian) where double-cropping hybrid rice is cultivated, 66 isolates from Central China rice-growing areas (Hubei, Hunan, South Henan, South Jiangsu, South Anhui, Jiangxi and Zhejiang) where both single-cropping and double-cropping systems are applied and the cultivars used include hybrid rice and japonica rice, 20 isolates from the Yunnan-Guizhou Plateau (Yunnan and Guizhou) which is a single- and double-cropping rice hybrid area and the main cultivar is hybrid rice but japonica rice is also cultivated on a small scale, and 16 isolates from the Northeast China rice-growing area (Liaoning) where single cropping japonica rice is cultivated.

### DNA extraction

Each isolate was cultured in 20 ml liquid potato-dextrose broth in a 100 ml conical flask at 26°C and was shaken at 200 rpm. After 7 days, the culture was transferred to 50 ml liquid potato-dextrose broth, in a 250 ml conical flask, and incubated under the same conditions for 3 days. The mycelia were harvested and immediately ground into a fine powder in liquid nitrogen for DNA extraction following the protocol described by Nakada et al. [30]. The DNA was precipitated with ethanol after two phenol/chloroform (1:1) extractions and one chloroform extraction. All isolates were confirmed as *U* *. virens* by PCR amplification using a pair of *U* *. virens* specific primers: US1-5/US3-3, which target the nrDNA ITS region [14].

### Molecular marker determination

In order to identify the genomic regions where SNPs are highly enriched, the genome sequences (unpublished data) of the two *U* *. virens* isolates: cce007 and csc086, were comparatively analyzed by Biolign software version 4.0.6 [31]. Isolate cce007 was collected from Hubei province of Central China rice-growing areas while isolate csc086 was collected from Sichuan province of Sichuan-shaanxi basin rice-growing areas. Since these two rice-growing areas are different in topography, climate, cropping regionalization and rice cultivars, genetic difference might exist between these two isolates. PCR primers, which were used to amplify 600–1000 bp DNA fragments in selected SNP-rich loci, were then designed using the online software, Primer3 (http://frodo.wi.mit.edu/primer3/). The SNP sites of each selected genomic locus were identified by aligning the sequences at each selected locus in 10 isolates from 10 different provinces using the DNAMAN software (Lynnon Corporation, Quebec, Canada). The putative functions of SNP-rich DNA fragments were deduced from BLAST alignment against sequences in GenBank (http://www.ncbi.nlm.nih.gov/) and gene prediction using GlimmerHMM software [32].

### Amplification and sequencing of DNA fragments

SNP-rich DNA fragments were amplified using specific primers (Table 1). Each reaction mixture was composed of a 1× PCR buffer (10 mM Tris-HCl, pH 8.3, 50 mM KCl, 1.5 mM MgCl_2_), 200 µM dNTP, 0.4 µM of each primer and 1 U of *Pfu* DNA polymerase (TaKaRa). The PCR was performed in a thermocycler (Labnet, TC9600; NJ, USA) under the following conditions: denaturation at 94°C for 2 min, followed by 30 cycles of denaturation at 94°C for 20 s, annealing at 56°C for 30 s and elongation at 72°C for 1 min. The PCR products were isolated by electrophoresis and purified for bi-directional sequencing.

**Table 1:** Primer pairs used for the SNP marker assays in this study and their nucleotide variations.

Fragment	Primers sequence (5′→3′)	Tm (°C)	Size of PCR products	Aligned length	No. of SNP sites	No. of alleles
F1	F: GGTCGGATACTCGGTGCC	58.0	853 bp	775 bp	30	16
	R: CGCTTAGGGCATCTTTCAC	55.8				
F2	F: GGTTCCGCTAGGGGCGATTG	66.3	776 bp	712 bp	25	9
	R: TGACGGGGGCGTAGTAAGTTT	61.4				
F3	F: TTGGCGGAGGAGATCAGGGTG	66.7	668 bp	594 bp	70	7
	R: TGCTGGTGGGAGGCGTTGA	65.2				

### Phylogenetic analysis

DNA sequences were aligned using Clustal X 2.1 software [33]. The resulting alignments were manually adjusted where necessary. Neighbor-joining (NJ) phylogenetic analysis was performed with the Kimura 2-parameter model using MEGA 5 software [34]. Maximum likelihood (ML) analysis was performed with the GTR+CAT model using the version RAxML v7.2.8 [35]. Bootstrap tests were based on 500 re-samplings and midpoint rooting was used for every data set. A bootstrap portion greater than 70% was considered significant. In addition, we tested for heterogeneity between the three markers using the Incongruence Length Difference test, implemented as the partition homogeneity test in PAUP (Sinauer Associates).

### Population genetics analysis

Population genetics analysis was performed within and between populations (i.e. the above mentioned five rice cropping regions) using concatenated DNA sequence data set at three genomic loci. For within-population genetic analysis, we calculated the haplotype diversity and analyzed linkage disequilibrium (1,000 randomizations) using MultiLocus 1.3 [36]. Evidence of recombination was examined by calculating the index of associations (*I*_A_ and *r*_*d*_) and by analyzing the allelic combinations. The number of haplotypes and nucleotide diversity were calculated using DnaSP software (version 5.10) [37]. For cross-population analysis, *F*_ST_ values were calculated using Arlequin 3.5.1.2 [38], based on variable nucleotides from the concatenated data set. Sources of genetic differentiation were investigated using analysis of molecular variance (AMOVA) tests, which were undertaken using GenALEx 6.5 [39]. Population structure was inferred using the Structure 2.3 program [40,41] with the default settings based on the concatenated data set and the result was visualized using Distruct 1.1 [42]. Principal coordinate analysis (PCA) was performed with individual isolates using GenALEx. The Mantel test was performed using GenALEx in order to examine the level of genetic isolation by geographical distances.

## Results

### Identification of SNP-rich genomic DNA markers

In order to analyze the genetic diversity and population structure of *U* *. virens* , we explored new DNA markers that contained multiple SNP sites. First, two *U* *. virens* isolates (cce007 and csc086) were chosen to do comparative analysis between their genome sequences. The assembled genome of cce007 is 38.8 Mb with a contig N50 length of 29.3 kb and the assembled genome of csc086 is 35.5 Mb with a contig N50 length of 27 kb (data not shown).

**Figure 2:**
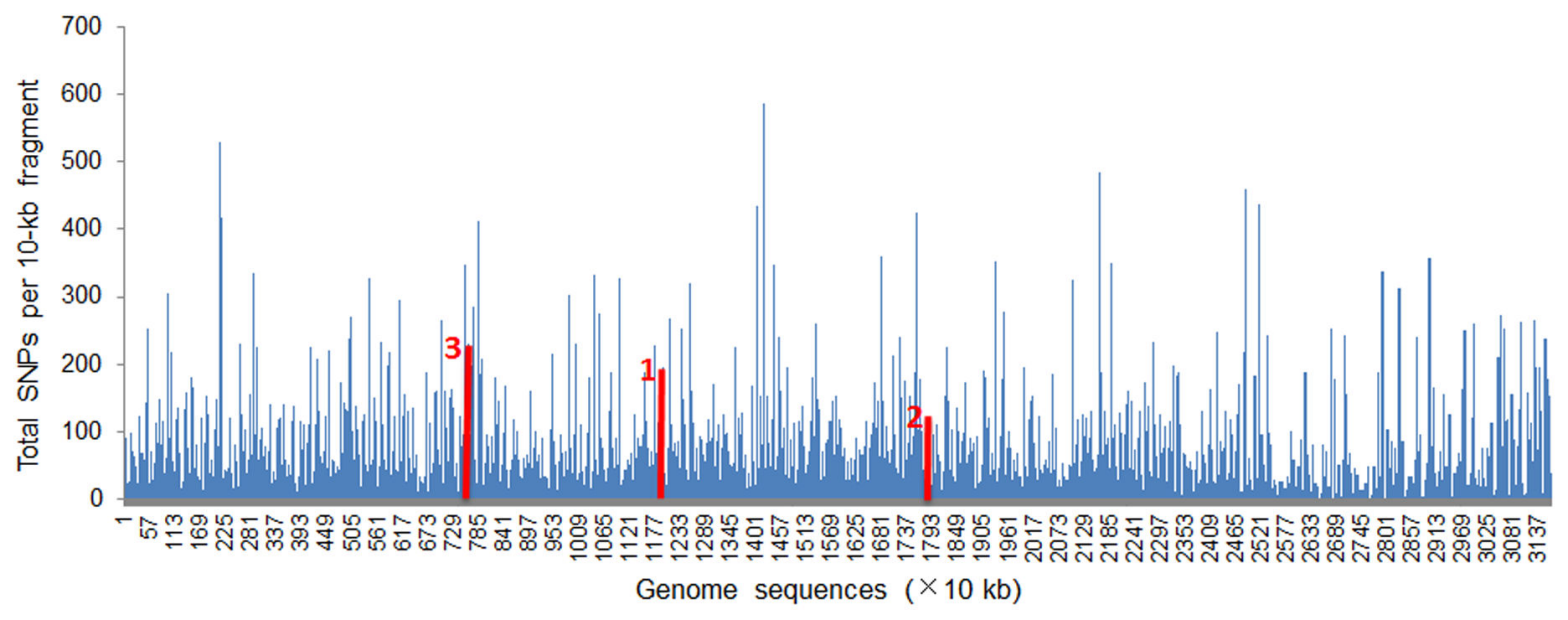
SNP sites comparison between the genome sequences of two *U. virens* isolates. The SNP numbers per 10 kb DNA were compared using two genomes sequences and Biolign software version 4.0.6 (Hall 2001). Locations of the three SNP-rich markers are indicated by red lines.

By comparing the genome sequences of two *U* *. virens* isolates, a total of 149669 SNP sites were found within 31.67 Mb matched genomic DNA between two isolates. As shown in Figure 2, the SNP sites were highly enriched in some regions. Ten genomic regions, each of which was approximately 700 bp in size and contained more than 10 SNP sites, were then randomly selected. The PCR products of each selected locus, from 10 isolates collected from 10 different provinces, were then sequenced and compared by alignment. Six genomic regions, from which PCR products were successfully amplified in all 10 isolates, were selected to generate PCR products in 162 isolates. As a result, only three genomic regions,that contain dense SNP sites and could be easily amplified by PCR in all isolates, were selected as DNA markers in order to analyze the genetic diversity and population structure of *U* *. virens* . For the other genomic regions, their PCR products could be obtained in some isolates but not in other isolates. The presence of SNP sites at the primer regions in some isolates might affect the efficacy of PCR application.

These DNA markers were named as Marker 1 (775 bp with 30 SNPs), Marker 2 (712 bp with 25 SNPs), and Marker 3 (594 bp with 70 SNPs) (Table 1). Gene prediction indicated that Marker 1 was a coding sequence without any introns, Marker 2 was a non-coding region and Marker 3 covered a coding region (50 bp at the 3′ end) and a non-coding region (544 bp at the 5′ end). A BLASTp search showed that the protein encoded by Marker 1 was homologous to a hypothetical protein (MGG_13116) from *Magnaporthe* *oryzae* . The nucleotide sequences of the three markers from the representative isolate, cce003, were deposited in GenBank under the following accession numbers: JX483852 for Marker 1, JX483853 for Marker 2 and JX483854 for Marker 3. Sequence variations in these markers among the *U* *. virens* isolates are shown in Table S2.

### Nucleotide variations of SNP-rich markers among *U* *. virens* isolates

A total of 162 *U* *. virens* isolates from 15 provinces, covering the five major rice-growing areas of China, were used to investigate the genetic diversity and population structure of the fungus (Figure 1; Table S1). The PCR products targeting the three SNP-rich DNA markers were successfully amplified from 162 isolates. Sequence analysis of the PCR products identified 30, 25 and 70 SNP sites and 16, 9 and 7 sequence types at Markers 1, 2 and 3, respectively. These SNP sites, 125 in total, included 118 parsimony informative sites and seven singleton variable sites. The parsimony informative sites included one insertion/deletion, 95 transitional substitutions, 19 transversional substitutions and three A/C/G substitutions (Table S2).

### Haplotypes within the 162 *U* *. virens* isolates

Analyses of the combined sequences at the SNP-rich DNA regions in the 162 isolates by software DnaSP version 5.10 [37] identified a total of 56 multilocus sequence types (haplotypes). Thirty-five haplotypes were represented by only one isolate and the remaining 21 haplotypes had at least two isolates (Table S2). Haplotypes 1, 10 and 11 were dominant and contained 30 (18.5% of total number of isolates), 15 (9.3%), and 15 (9.3%) isolates, respectively (Table S2).

Seven haplotypes, each of which contained at least two isolates, were only found in one area. For instance, all five isolates of haplotype 42 were collected from the Sichuan-Shaanxi basin rice-growing areas, and all three isolates of haplotype 6 were collected from Central China (Table S2). However, 12 haplotypes were shared by three or more areas. Haplotype 1 and haplotype 17 were found in all five areas.

### Phylogenetic analysis

**Figure 3:**
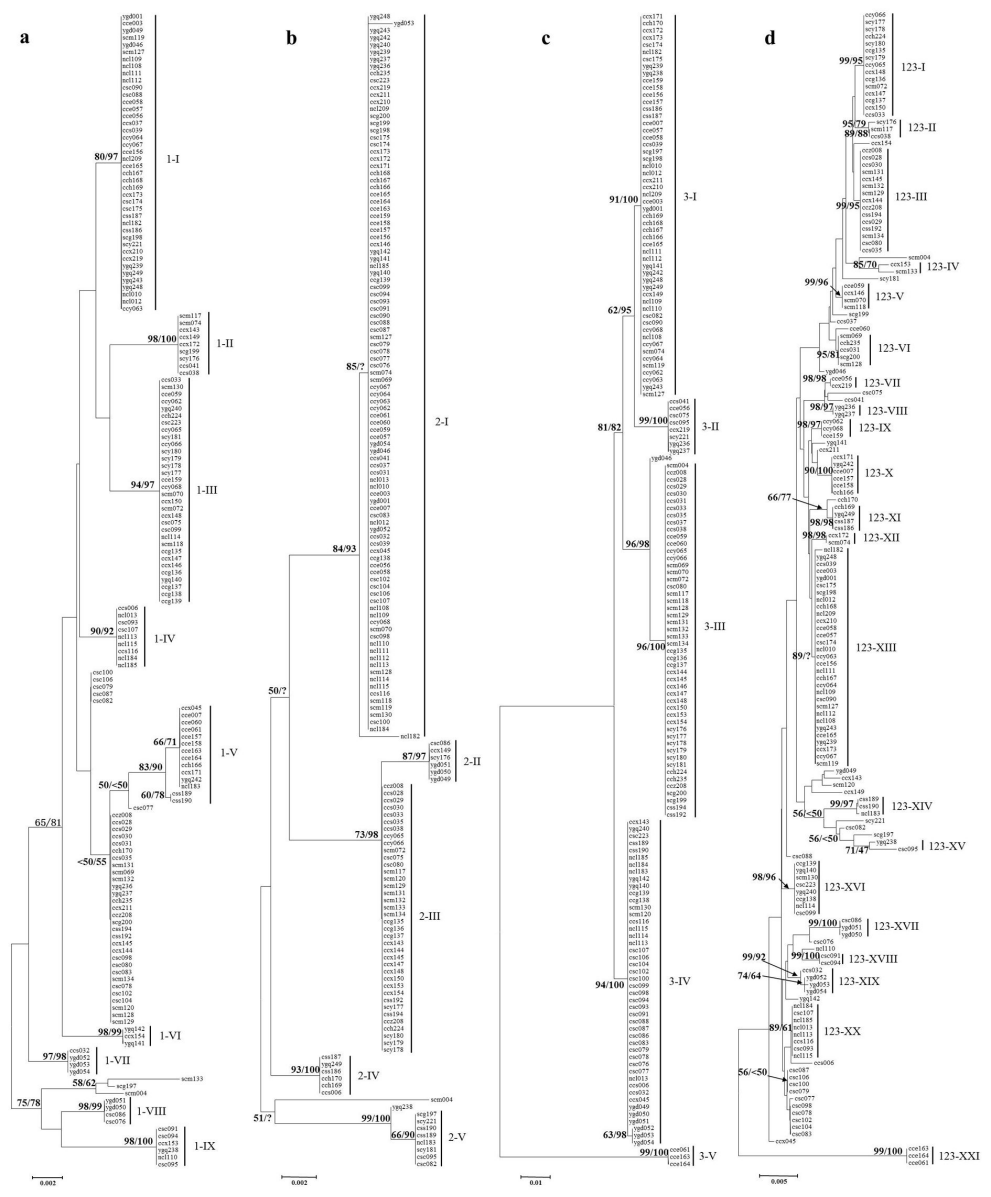
Phylogenetic analyses based on sequences at individual marker (a–c) or the combined sequences at three markers (d). NJ and ML phylogenetic analyses were conducted based on DNA sequences at Marker 1(a), Marker 2 (b), Marker 3 (c) and the combined sequences at the three markers (d). The two methods gave almost identical topologies. Nodal support values for NJ (before the slash) and ML analysis (after the slash) are given for branches receiving more than 70% support. Clades were named with marker names and Roman numbers.

Three phylogenetic trees were built using the DNA sequences of the 162 isolates found in each individual SNP-rich region. As shown in Figure 3A–C, the 162 isolates could be grouped into 9, 5 and 5 clades, based on sequences at Marker 1, Marker 2 and Marker 3, respectively. Most of the clades contained isolates from different rice cropping regions. The partition homogeneity test revealed that the phylogenetic analysis results, based on sequences at individual SNP-rich markers, strongly conflict with each other (*P* = 0.001). The 162 isolates could be grouped into more than 20 clades, based on the combined sequences at the three SNP-rich markers, and the isolate numbers for most clades were less than five (Figure 3D).

### Population genetic structures

The 162 *U* *. virens* isolates used in this study were grouped into five geographical populations, each of which had 6–26 haplotypes. As shown in Table 2, with the exception of Northeast China, most of these populations displayed high nucleotide diversity and haplotype diversity. Central China possessed the highest genetic variation. The linkage disequilibrium analysis revealed that recombination occurred within most of the populations when all the populations were considered as a whole (Table 2). Genetic recombination occurred amongst all possible locus pairs (Figure S1). False smut pathogen has sexual stage because it produces sclerotia from which fruiting bodies form. When two compatible strains meet, their hyphae might fuse and the recombination might take place between two strains. However, the formation of fruiting bodies in fields has not previously reported in *U* *. virens* . To verify its genetic recombination revealed by this SNP-rich marker-based study, it needs to find the sexual stage of this fungus in fields first.

**Table 2:** Genetic variation patterns within populations.

Population	No. of sample	No. of haplotypes (Frequency of the most common haplotype)	Nucleotide diversity,*Pi* (×10^−2^)	Haplotype diversity	*I*_A_	*r_d_*	PcP
Sichuan-Shaanxi basin	32	16 (5)	0.75	0.95	0.44** (0.27*)	0.23** (0.15*)	0.00
Yunnan-Guizhou Plateau	20	13 (4)	0.79	0.95	0.19* (−0.11)	0.09* (−0.06)	0.66
South China	28	16 (5)	0.82	0.94	0.25** (−0.05)	0.13** (−0.03)	0.00
Central China	66	26 (13)	0.98	0.92	0.50** (−0.03)	0.26** (−0.02)	0.00
Northeast China	16	6 (7)	0.49	0.74	0.56** (−0.05)	0.28** (−0.03)	1.00
Total	162	56 (30)	0.93	0.94	0.38** (−0.02)	0.19** (−0.01)	0.00

Note: *I*_A_, index of association; *r*_*d*_, scaled index of association (*I*_A_) by the number of loci (m-1) and PcP, percentage of phylogenetically compatible pairs of loci. *I*_A_ and *r*_*d*_ values were estimated for both uncorrected (outside parentheses) and clone-corrected (within parentheses) data sets. * *P* < 0.05; **P < 0.01.

**Figure 4:**
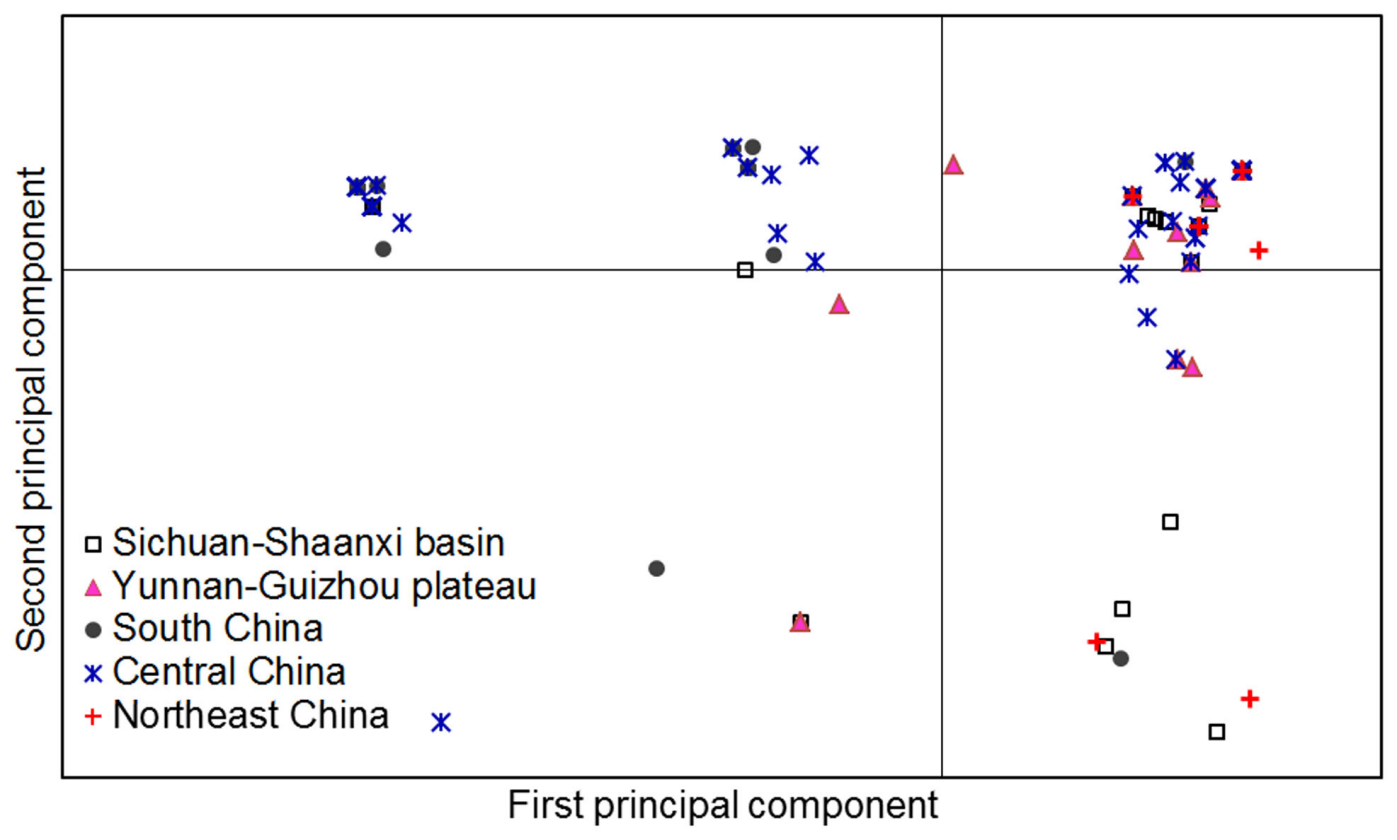
Principal component analysis (PCA) for 162 individual isolates from five major rice-growing areas in China. Individuals within the same population are marked using the same symbols. The first and second principal coordinates account for 41.6% and 23.0% of the variation, respectively. Note that individuals from a population may have separated and gathered with individuals from another population.

**Figure 5:**
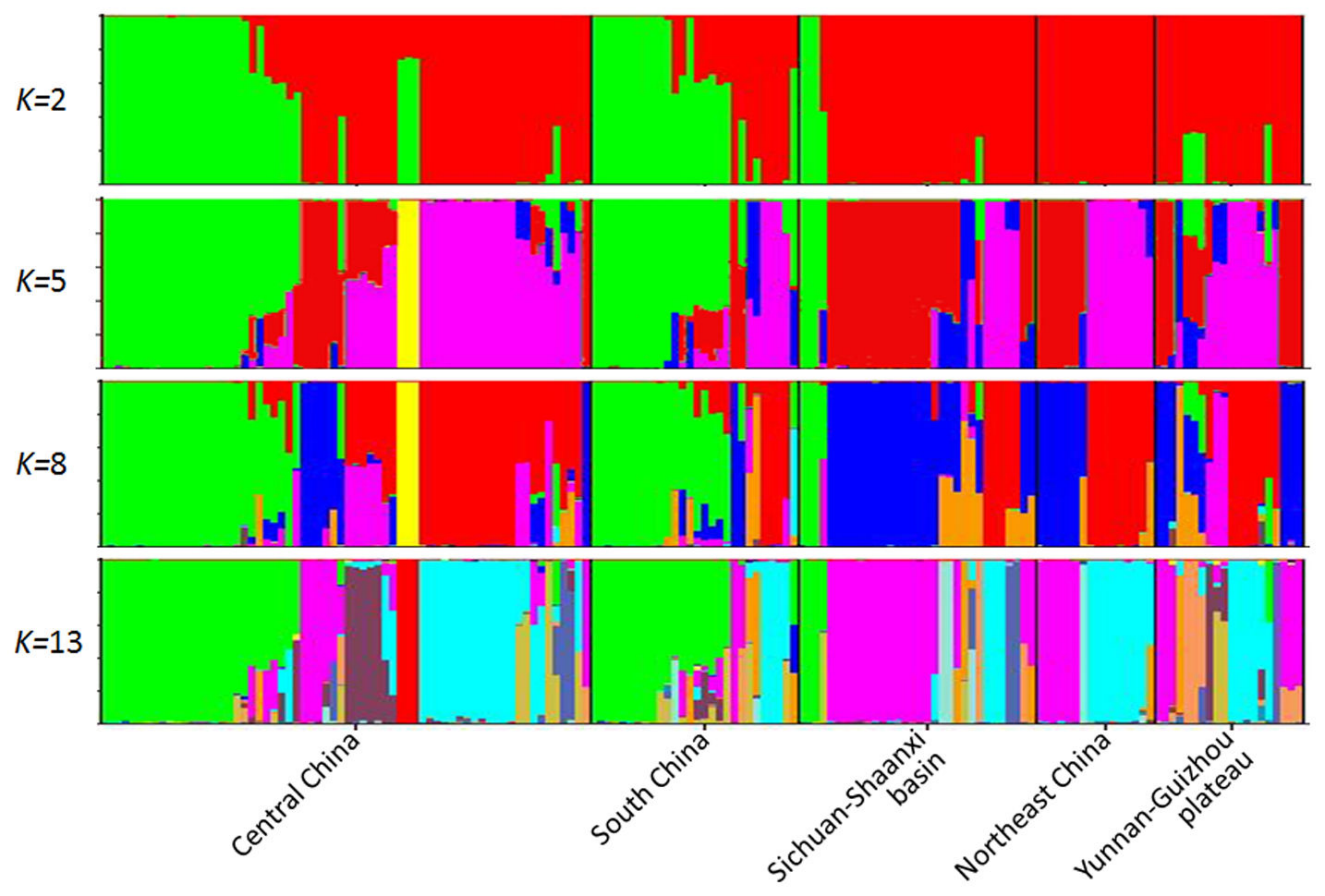
Population structure of *U. virens* inferred from 125 SNPs of 162 isolates using the program Structure (version 2.3.1), with different *K* values. An admixture model with correlated allele frequencies was used. Each isolate is represented by a single vertical line broken into *K* colored segments, with lengths proportional to each of the *K* inferred clusters.

**Figure 6:**
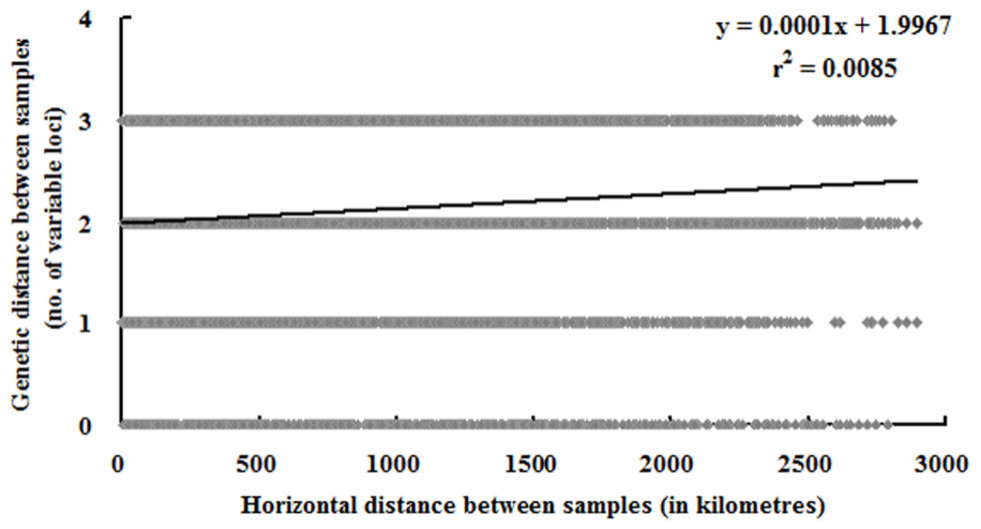
Mantel test between genetics and horizontal distance. Horizontal geographical distances means distances based on longitudinal–latitudinal coordinates.

When two populations were compared, significant genetic differentiation could be found in most of the compared pairs, based on *F*_ST_ values. The only two exceptions were the pairs: Yunnan-Guizhou Plateau versus Sichuan-Shaanxi basin and Yunnan-Guizhou Plateau versus Northeast China (Table 3). AMOVA analysis found that the difference between these populations contributed to 11% of the total genetic variation while 89% of the genetic variation came from within individual populations. This is consistent with the fact that several haplotypes were shared by samples from different populations (Table S2), which indicated that gene flow occurred between populations. Genetic differences among isolates within populations and gene flow between different populations were also verified by principal component analysis (Figure 4) and model-based clustering algorithm analysis by STRUCTURE (Figure 5). Furthermore, a significant and positive correlation (*P* = 0.001) between genetic and geographic distances was detected with a correlation coefficient (r) of 0.09 (Figure 6). Further investigations found that the genetic distances were significantly correlated with longitude gradients (*r* = 0.08; *P* = 0.01), but not with latitude gradients (r = 0.01; *P* = 0.30).

**Table 3:** Pairwise *F*_ST_ values between different populations.

Population	Sichuan-Shaanxi basin	Yunnan-Guizhou Plateau	South China	Central China
Yunnan-Guizhou Plateau	0.02			
South China	0.24***	0.24***		
Central China	0.12***	0.09***	0.06**	
Northeast China	0.08*	0.02	0.34***	0.13***

* *P* < 0.05; **P < 0.01; ***P < 0.001.

## Discussion

Sequence-based markers are important in both molecular systematic and population genetic analyses because they can provide unambiguous information on defined loci and enable the direct assessment of variability [43]. In this study, three SNP-rich DNA markers, located in three different genomic regions, were identified by comparing the genomic sequences of two *U* *. virens* isolates and this was further confirmed by the sequence analysis of 10 isolates from 10 different provinces. Most of the DNA sequences used for MLST in other fungi are housekeeping genes [23–26]. However, none of the SNP-rich DNA markers found in this study were housekeeping genes. Therefore, this study provided novel DNA markers for analyzing the genetic diversity of fungal species, especially for species with poor polymorphism in commonly used housekeeping genes. In addition, these defined markers can be used to identify field *U* *. virens* isolates in the future. As far as can be ascertained, this is the first report on the DNA-sequence-based analysis of the genetic diversity and population structure of *U* *. virens* .

Analysis and comparison of the sequences of these SNP-rich regions revealed that *U* *. virens* populations in China have at least 56 multilocus sequence types (haplotypes). This means that *U* *. virens* isolates in the field are genetically diverse. However, the correlation between virulence and genotype is unknown. Although several haplotypes, such as haplotype 1 and 17, were widely distributed over the rice-growing areas, the genetic differentiations between isolates from geographically distant rice-growing areas were higher than isolates between adjacent rice-growing areas. In addition, the *U* *. virens* isolates collected from similar regions displayed considerable DNA composition stability. These results suggested that, naturally, *U* *. virens* in China might not be spread over long distances. The wide distribution of some *U* *. virens* haplotypes might be due to the transportation of rice seeds contaminated with smut balls containing chlamydospores.

This study demonstrates that the genetic diversities of *U* *. virens* populations are high in most of rice-growing areas. The 162 isolates could be grouped into more than 20 clades. The high genetic diversity in *U* *. virens* might be caused by genetic recombination through sexual cross between mating compatible strains in fields. In laboratory condition, *U* *. virens* has sexual stage during which fruiting bodies emerge from sclerotia and ascospores are released [1]. Our data clearly suggest that a sexual stage in fields does take place and does contribute to the genetic diversity of *U* *. virens* populations. The genetic diversity of *U* *. virens* in Northeast is relatively lower than other areas. Similar to this, an AFLP-based analysis showed that the genetic variation *U* *. virens* populations of Liaoning province were low [20]. The lower genetic divergence of *U* *. virens* in Northeast suggests that sexual reproduction in this region might be less active than other regions. Climate conditions might affect sexual reproduction of *U* *. virens* .

## Supporting Information

Table S1 **Geographical distributions and haplotypes of *U. virens* isolates.**
(XLS)

Table S2 **Sequence differences among 56 haplotypes found at the three loci with enriched SNP sites.**
(XLS)

Figure S1 **Allelic combination showing evidence of recombination.**
(DOC)
