# Correction: Genetic Diversity and Population Structure of Rice Pathogen *Ustilaginoidea* *virens* in China

**DOI:** 10.1371/annotation/13aec20c-1d9d-4c18-a9a3-a8767987b9b8

**Published:** 2013-12-05

**Authors:** Xianyun Sun, Shu Kang, Yongjie Zhang, Xinqiu Tan, Yufei Yu, Haiyong He, Xinyu Zhang, Yongfeng Liu, Shu Wang, Wenxian Sun, Lei Cai, Shaojie Li

This paper was republished on Decemeber 4 due to two figures being merged in the PDF of the article. Please download the corrected PDF from the online article. 

