# Supplementary material for: Genetic Diversity and Population Structure of Rice Pathogen Ustilaginoidea virens in China
Source: PLoS One. 2013 Sep 30;8(9):e76879. doi: 10.1371/journal.pone.0076879 (PMC3786968; doi:10.1371/journal.pone.0076879)
Supplement: Figure S1 — Allelic combination showing evidence of recombination. (DOC) [file pone.0076879.s003.doc]

**Figure S1.** Allelic combination showing evidence of recombination. Allele identifiers of a locus are listed above the horizontal line or on the left side of the vertical line. The number of samples of each allelic combination is shown below the horizontal line and on the right side of the vertical line.
